# The use of automated pupillometry to assess cerebral autoregulation: a retrospective study

**DOI:** 10.1186/s40560-020-00474-z

**Published:** 2020-07-31

**Authors:** Armin Quispe Cornejo, Carla Sofía Fernandes Vilarinho, Ilaria Alice Crippa, Lorenzo Peluso, Lorenzo Calabrò, Jean-Louis Vincent, Jacques Creteur, Fabio Silvio Taccone

**Affiliations:** Department of Intensive Care Medicine, Erasme University Hospital, Route de Lennik, 808, 1070 Brussels, Belgium

**Keywords:** Cerebral autoregulation, Pupillometry, Sepsis, Brain monitoring

## Abstract

**Background:**

Critically ill patients are at high risk of developing neurological complications. Among all the potential aetiologies, brain hypoperfusion has been advocated as one of the potential mechanisms. Impairment of cerebral autoregulation (CAR) can result in brain hypoperfusion. However, assessment of CAR is difficult at bedside. We aimed to evaluate whether the automated pupillometer might be able to detect impaired CAR in critically ill patients.

**Methods:**

We included 92 patients in this retrospective observational study; 52 were septic. CAR was assessed using the Mxa index, which is the correlation index between continuous recording of cerebral blood flow velocities using the transcranial Doppler and invasive arterial blood pressure over 8 ± 2 min. Impaired CAR was defined as an Mxa > 0.3. Automated pupillometer (Neuroptics, Irvine, CA, USA) was used to assess the pupillary light reflex concomitantly to the CAR assessment.

**Results:**

The median Mxa was 0.33 in the whole cohort (0.33 in septic patients and 0.31 in the non-septic patients; *p* = 0.77). A total of 51 (55%) patients showed impaired CAR, 28 (54%) in the septic group and 23 (58%) in the non-septic group. We found a statistically significant although weak correlation between Mxa and the Neurologic Pupil Index (*r*^2^ = 0.04; *p* = 0.048) in the whole cohort as in septic patients (*r*^2^ = 0.11; *p* = 0.026); no correlation was observed in non-septic patients and for other pupillometry-derived variables.

**Conclusions:**

Automated pupillometry cannot predict CAR indices such as Mxa in a heterogeneous population of critically ill patients.

## Background

Critical illness is characterized by an acute and severe impairment in vital functions, requiring the admission to the intensive care unit (ICU) to receive specific therapies and an adequate support to the failing organs. Among all the potential causes of ICU admission, sepsis or septic shock is one of the most common and causes millions of deaths and long-term disabilities among survivors each year [1–4]. Multiple studies have shown evidence of neurological impairment in critically ill patients, in particular during sepsis, despite the absence of intracranial source of infection or the demonstration of a primary brain injury [5]. Blood-brain barrier dysfunction, neurotransmission abnormalities, neuroinflammation, as well as alterations of the brain’s microcirculation have been described in this setting [6–8]. However, some autoptic studies have shown signs of cerebral ischemia, thus suggesting a role for cerebral hypoperfusion in the development of neurological dysfunction in critically ill patients [9].

One of the mechanisms that could promote brain hypoperfusion in this setting is the impairment of cerebral autoregulation (CAR), i.e., the capacity to maintain an adequate and stable cerebral blood flow (CBF) in response to different systemic stimuli, such as changes in arterial pressure of carbon dioxide (PaCO_2_), or to local stimuli, such as metabolic activation of the different brain areas. Impaired CAR has been described in brain-injured patients [10, 11]. Furthermore, impaired CAR was associated with the development of sepsis-associated brain dysfunction (SABD) and long-term cognitive impairment after sepsis resolution [5, 12–14].

Much remains unknown about the physiology of CAR, its assessment, and the most effective clinical interventions to optimize patient outcome. One of the most common evaluations of CAR is the interaction between changes in CBF as response to changes in cerebral perfusion pressure (CPP) or mean arterial pressure (MAP); thus, if the autoregulatory system is overridden, CBF becomes pressure-dependant [15, 16]. Importantly, the effectiveness of the mechanisms involved in CAR (i.e., myogenic, neurogenic, metabolic) also depends on the interaction with the autonomic nervous system (ANS), which can influence the diameter and the reactivity of intracranial blood vessels via different efferent pathways [16–18]. As such, available indices of CAR function, such as the Mxa index, which is calculated using CBF velocities and MAP [15], may have some correlation with variables assessing ANS function, in particular in critically ill patients, with various alterations of both systems being reported.

Autonomous nervous system dysfunction is evaluated in critically ill patients using different approach, including the heart rate variability; however, pupillary size and pupillary light reflex (PLR) [19], which are routinely assessed in this setting and can be nowadays quantified at the bedside using automated pupillometers [20–22], may also give some information on ANS. In particular, the neurological reflex loop to light stimulus is partially controlled by ANS, i.e., the parasympathetic system through the third cranial nerve and the ascending sympathetic pathways of the cervical spinal nerves [21, 23–25]; as such, considering the potential relationship between CAR, ANS, and pupillary function, one may wonder whether CAR could be quantified using the assessment of PLR.

The aim of this study was therefore to evaluate, whether the PLR assessed by the automated pupillometer could detect impairment of CAR in critically ill patients.

## Methods

### Study design and population

We retrospectively analyzed all critically ill adult (> 18 years) patients admitted to the intensive care unit of the Erasme Hospital between January 2017 and March 2019. Patients were eligible if they underwent concomitantly CAR assessment and PLR analysis via an automated pupillometry. Both techniques were performed according to the availability of the devices and to the patient’s condition at the medical round and considered within the common practice. To avoid the effects of long-term ICU stays on the interpretation of the data, only patients admitted since less than 48 h in the ICU were considered. Results of CAR assessment and pupillometry were prospectively reported in the patient data management system (PDMS). Exclusion criteria were pre-existing ocular disease or surgery, previous central nervous system disorders, arrhythmias, being under ECMO therapy, having supra-aortic arteriopathies or the absence of an arterial line for continuous MAP recordings. The local ethics committee approved the study protocol (reference P2019/204) and waived the need for the informed consent.

### Data collection

We collected demographic data, comorbid diseases (i.e., chronic kidney disease, heart failure, diabetes, chronic respiratory diseases) as well as the Acute Physiology and Chronic Health Evaluation (APACHE) II score on admission. The presence of sepsis, according to standard Sepsis-3 definitions [1], was recorded. Data on the concomitant therapies (i.e., mechanical ventilation or vasopressors) as well as arterial blood gases at the time of the CAR and pupillometry assessment were also collected. Moreover, some biological and hemodynamic data on the day of the data collection were reported.

### Automated pupillometry

Pupillary light reflex was performed using an automated pupillometer, the NeurOptics NPi-200 instrument (Neuroptics, Irvine, CA 92612, USA), which uses an infrared camera that integrates a calibrated light stimulation of fixed intensity (1000 Lux) and duration (3.2 s) to provide rapid measurement (0.05 mm limit) of pupil size and quantitative PLR (i.e., the difference between baseline and post-stimulation pupil size, expressed as percentage of constriction from the baseline value), constriction velocity, and latency. The measurement is completed in less than 30 s for each eye, and a minimum duration of 1 min was allowed between appraisals of the two pupils to obtain full recovery of baseline pupil diameter after light stimulation. Median values from both eyes were used for comparison with CAR findings. Based on an integrated algorithm, the automated pupillometry calculates the Neurological Pupil Index (NPI), as an index of normal (NPI > 3) vs. abnormal (NPI 0–3) pupillary function. The NPI has the advantage of being minimally influenced by external factors, and in particular is unaffected by opioids and variable individual baseline pupil size [26, 27]. The pupillary evaluation was performed in complete darkness concomitantly to the CAR evaluation with the TCD, as a standard procedure in the ICU.

### Transcranial Doppler and cerebral autoregulation assessment

Blood pressure (BP) was zeroed at the level of the right atrium (Baxter Healthcare Health Care Corp. Cardio Vascular Group, Irvine, CA); no correction was made for hydrostatic pressure influence. Transcranial Doppler (TCD) was used to assess blood flow velocity (FV) in the left middle cerebral artery (MCA) using the Doppler Box (DWL Compumedics, Singen, Germany); one operator (AQC) performed all TCDs. All recorded signals were digitized via an A/D converter (DT9801; Data Translation, Marlboro, MA), sampled with a frequency of 50–100 Hz. After a methodological removal of artifacts, we used a custom-written script (MATLAB release 2015b, MathWorks, USA) to assess autoregulation: BP and FV were then averaged on a 10-s moving window with 50% overlap, and the Pearson correlation coefficient between BP and FV was calculated. The Pearson correlation coefficient between BP and FV is called mean flow index (Mxa). The Pearson correlation coefficient (*r*) represents the strength and direction of a relationship between two variables. It has a value between + 1 and − 1, where 1 represents a total positive correlation, 0 represents no correlation, and − 1 represents total negative correlation. The correlation is considered to be moderately positive when *r* > 0.3. Given that changes in FV mirror changes in CBF, Mxa > 0.3 means that CBF is dependent on BP changes, and autoregulation is “impaired”; when BP and FV have a negative or weak correlation (Mxa ≤ 0.3), autoregulation is considered intact [15, 28–30].

### Statistical analysis

Data are expressed as median (25th–75th percentiles) or count (percentage). Comparisons between “impaired” and “intact” CAR with variables from the pupillometer were performed using a Wilcoxon rank test for continuous variables. Comparisons within groups (i.e., sepsis vs. non-septic) were also performed. The Pearson correlation coefficient, or Pearson’s *r*, was used to measure the linear correlation between Mxa and pupillometer-derived variables. The discriminative ability of pupillometry-derived variables to predict impaired cerebral autoregulation was evaluated using receiver operating characteristic (ROC) curves with the corresponding area under the curve (AUC) and related sensitivity and specificity. Statistical analyses were performed using GraphPad PRISM version 5.0 (San Diego, CA, USA). For all statistical tests, a *p* < 0.05 was considered significant.

## Results

### Study population

On a total of 123 patients undergoing CAR assessment over the study period, 92 had also concomitant pupillometry evaluation and were included in the final analysis. Characteristics of the study population are shown in Table 1; overall ICU mortality was 9%. Fifty-two patients (56%) were diagnosed with sepsis. The primary site of infection was abdominal (48.1%) and respiratory (21.2%). The most common cause of infection was bacterial (65%), in particular Gram-negative bacteria (50%). Septic patients had a higher APACHE II score on admission and had more frequently chronic renal failure, heart failure, and obesity than others (Table 1). Also, septic patients presented more frequently with encephalopathy on the day of CAR assessment and received more often both anti-hypertensive and vasopressors than non-septic patients. Mortality was higher, although not statistically significant, in septic than in non-septic patients.

**Table 1:** Population demographics and statistical analysis in the septic and non-septic groups

	All (*n* = 92)	Septic (*n* = 52)	Non-septic (*n* = 40)	*p* value
Age, years	62 [58–67]	62 [54–70]	62 [60–65]	0.10
Male sex, %	68.5	71.2	65	0.53
APACHE II score	19 [11–28]	22 [16–28]	7 [1–13]	< 0.001
ICU LOS, days	5 [3–8]	6 [3–8]	5 [2–7]	0.47
Alive at ICU discharge, %	91.2	86.5	97.4	0.07
Comorbidities				
CKD, %	19.6	28.8	7.5	0.01
Arteriopathy, %	30.4	38.5	20	0.06
CHF, %	16.3	7.7	27.5	0.01
COPD, %	15.2	15.4	15	0.96
Liver disease, %	30.4	32.7	27.5	0.59
Diabetes mellitus, %	37	32.7	42.5	0.33
Chronic anemia, %	14.1	19.2	7.5	0.11
Smoking, %	16.3	17.3	15	0.77
Alcohol consumer, %	22.8	26.9	17.5	0.29
Obesity, %	18.5	26.9	7.5	0.02
Encephalopathy, %	28.3	36.5	17.5	0.04
At time of assessment				
Sedation, %	20.7	21.2	20	0.89
Analgesia, %	47.8	46.2	50	0.71
NMBA, %	3.3	5.8	0	0.12
Mechanical ventilation, %	34.8	36.5	32.5	0.69
Antihypertensives, %	32.6	21.2	47.5	0.01
Norepinephrine, μg/min	24.4 [14.1–34.6]	29.1 [0.1–58.1]	2.6 [−0.4–5.6]	< 0.001
Dobutamine, %	10.9	11.5	10	0.81
MAP, mmHg	77 [71–84]	77 [71–83]	80 [70–89]	0.83
Heart rate, beats/min	95 [82–109]	96 [82–110]	93 [75–111]	0.03
Temperature, °C	37 [36.6–37.6]	37.1 [36.7–37.4]	37.4 [36.9–37.9]	0.78
Hemoglobin, g/dL	9.9 [8.3–11.4]	9.8 [8–11.5]	10.5 [9.3–11.8]	1.00
Hematocrit, %	29.3 [24.2–34.5]	29 [23.5–34.5]	31 [27.2–34.9]	0.86
C-reactive protein, mg/L	215 [116–314]	234 [139–329]	127 [2–252]	< 0.001
Sodium, mmol/L	136.6 [134.7–138.5]	136.8 [134.3–139.3]	135.6 [134.6–136.6]	0.34
PEEP, cmH_2_O	5 [1–9]	5 [0.17–10]	2 [−0.5–5]	0.21
FiO_2_, %	39 [29–49]	39 [29–49]	35 [26–45]	0.72
pH	7.40 [7.35–7.46]	7.40 [7.34–7.45]	7.42 [7.35–7.49]	0.77
PaCO_2_, mmHg	37 [32–42]	37 [32–43]	37 [31–43]	0.02
PaO_2_, mmHg	89 [72–106]	87 [68–105]	98 [84–113]	0.28
P/F	264 [177–351]	253 [164–342]	315 [185–444]	0.72
Lactates	1.84 [1.19–2.49]	1.82 [1.22–2.42]	1.94 [1.19–2.69]	0.19
Mxa	0.33 [0.09–0.57]	0.33 [0.08–0.58]	0.31 [0.04–0.59]	0.77
Altered CAR, %	55.4	53.8	57.5	0.73
NPi	4.33 [3.98–4.69]	4.36 [3.98–4.73]	4.22 [3.75–4.7]	0.59
Pupil size, mm	3.67 [2.69–4.65]	3.56 [2.68–4.45]	3.81 [2.77–4.85]	0.32
Pupil constriction, %	32.52 [25.57–39.47]	32.20 [25.25–39.15]	32.93 [26.48–39.38]	0.84
Constriction velocity, mm/s	3.61 [3.07–4.15]	2.01 [1.47–2.55]	5.69 [5.14–6.25]	0.91
Latency, ms	0.24 [0.21–0.26]	0.24 [0.21–0.26]	0.24 [0.22–0.27]	0.22
Primary site of infection, %				
Abdominal	28.3	48.1	2.5	< 0.001
Respiratory	19.6	21.2	17.5	0.45
Urinary tract	6.5	11.5	0	0.03
Soft tissue	6.5	9.6	2.5	0.17
Blood/CVC	1.1	1.9	0	0.38
Unknown	2.2	3.8	0	0.21
Pathogen, %				
Bacterial	45.7	65.4	20	< 0.001
GNB	33.7	50	12.5	< 0.001
GPC	13	17.3	7.5	0.07
Fungus	5.4	5.8	5	0.87
Virus	4.3	3.8	5	0.79
Unknown bug	14.1	23.1	2.5	0.03
Other bug	1.1	1.9	0	0.38

*APACHE II* Acute Physiology, Age, Chronic Health Evaluation II, *ICU LOS* ICU length of stay, *CKD* chronic kidney disease, *CHF* chronic heart failure, *COPD* chronic obstructive pulmonary disease, *NMBA* neuro-muscular blocking agents, *PEEP* positive end expiratory pressure, *PaO*_*2*_ arterial oxygen pressure, *PaCO*_*2*_ arterial carbon dioxide pressure, *P/F* PaO_2_ divided by FiO_2_, *Mxa* mean flow index, *CAR* cerebral autoregulation, *NPi* Neurological Pupil Index, *CVC* central venous catheter, *GNB* gram-negative bacillus, *GPC* gram-positive cocci

### Cerebral autoregulation and pupillometry

On the day of CAR assessment, 20% of the patients were sedated, and 48% received analgesic drugs. The median Mxa index was 0.33 in the whole population; 51 (55%) patients had impaired CAR. Mxa was similar between septic and non-septic patients, with a similar number of patients having impaired CAR (28/52, 54% vs. 23/40, 58%; *p* = 0.72) in the two groups. Median NPI and pupil size were 4.3 and 3.7 mm, respectively; both values were similar between septic and non-septic patients, as for other pupillometry-derived variables (Table 1). No patient had unreactive pupils.

### Correlation between cerebral autoregulation and pupillometry

**Fig. 1:**
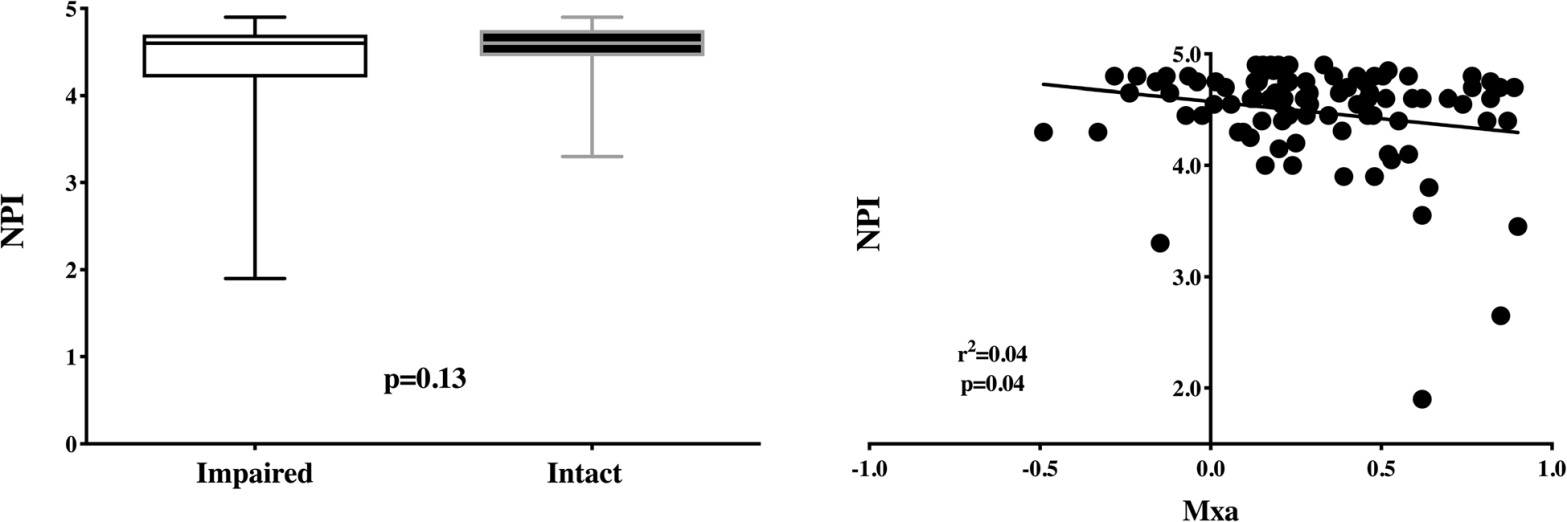
NPI values between patients with impaired and intact autoregulation (left); correlation between Mxa and NPI in the general cohort (right). Mxa, Mean flow index; NPI, Neurologic Pupil Index

The correlation analysis between NPI and Mxa was weak (*r*^2^ = 0.04), although statistically significant (*p* = 0.048—Fig. 1). All the other pupillometry-derived variables had no correlation with Mxa (Table 2). However, NPI values were similar between patients with impaired and intact CAR (4.6 [4.2–4.7] vs. 4.6 [4.5–4.8]; *p* = 0.13—Fig. 1). As such, the ROC curve analysis showed an AUC for NPI to predict impaired CAR of 0.59 [0.47–0.71].

**Table 2:** Pearson’s correlation between Mxa and pupillometry-derived variables in all patients

	All patients (*n* = 92)		Septic patients (*n* = 52)		Non-septic patients (*n* = 40)	
	***r***^***2***^	***p*** value	***r***^***2***^	***p*** value	***r***^***2***^	***p*** value
Mean NPI	0.04	0.048	0.11	0.03	0.01	0.602
Mean size, mm	< 0.0001	0.989	0.001	0.91	0.001	0.92
Mean CH, %	0.01	0.252	0.03	0.15	0.001	0.93
CV, mm/s	< 0.001	0.794	0.002	0.73	0.002	0.74
Latency, ms	< 0.001	0.832	0.02	0.33	0.04	0.21
Worst NPI	0.04	0.044	0.08	0.03	0.015	0.45

*NPi* Neurological Pupil Index, *CH* constriction rate, *CV* constriction velocity

**Fig. 2:**
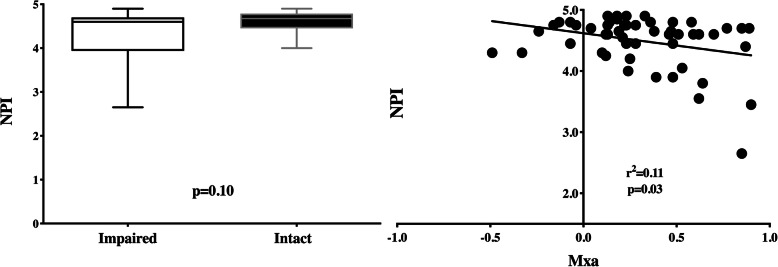
NPI values between septic patients with impaired and intact autoregulation (left); correlation between Mxa and NPI in septic patients (right). Mxa, Mean flow index; NPI, Neurologic Pupil Index

In the septic population, the correlation analysis between NPI and Mxa was weak (*r*^2^ = 0.11), although statistically significant (*p* = 0.03—Fig. 2). All the other pupillometry-derived variables had no correlation with Mxa (Table 2). However, NPI values were similar between patients with impaired and intact CAR (4.6 [3.9–4.7] vs. 4.7 [4.5–4.8]; *p* = 0.10—Fig. 2). As such, the ROC analysis showed an AUC for NPI to predict impaired CAR of 0.63 [0.48–0.78].

## Discussion

In this study, we observed only a weak correlation between NPI (i.e., one pupillometry-derived variable) and the Mxa, which is an index assessing CAR. This correlation remained significant in the septic patients’ group, while no correlation was observed in non-septic patients. Finally, NPI had limited predictive accuracy to identify impaired CAR.

The assessment of CAR has been growing in clinical practice, in particular to optimize cerebral perfusion pressure and prevention of secondary brain injury in different acute intracranial conditions, such as traumatic brain injury (TBI), subarachnoid hemorrhage (SAH), ischemic stroke, and post-anoxic encephalopathy [31–36]. Cerebral autoregulation is a complex, dynamic phenomenon which is hardly described in all its aspects by a simple mathematical model. The best technique to explore CAR is still uncertain, and new models are continuously developed in the effort to understand autoregulation physiology and to apply autoregulation monitoring to clinical practice [15, 37, 38]. Assessment of FV is one of the most reliable, less invasive techniques to assess cerebral autoregulation. Even if an absolute correlation between FV values and CBF values cannot be established, modifications in FV mirror modifications in CBF assuming that the diameter of the insonated vessel does not change over time, as it has been demonstrated in large intracranial vessel [15, 39]. However, autoregulation is not an “on-off” phenomenon, and a “grey zone” of uncertainty of autoregulation efficiency has already been described [33, 37]. Furthermore, although cerebral autoregulatory response is rapid, it is not instantaneous. The time course of development of the cerebral autoregulatory response is uncertain, as it is the threshold which correctly identifies intact CAR in clinical practice [40]. This uncertainty might explain the lack of surrogated to assess CAR using alternative tools; also, as CAR is also influenced by PaCO_2_, temperature, and comorbid diseases [41–43], many confounders could impact on Mxa without specifically modifying the pupillary response.

Pupillary size is controlled by the balance between sympathetic and parasympathetic systems, integrated at the midbrain level, as well as by neuronal activity of the locus coeruleus, colliculi, and cingulate cortex [23]. Multiple neurotransmitter systems have been identified to be involved in the control of cortical activity, which may also affect pupillary size, in particular acetylcholine and norepinephrine [23, 24]. Automated pupillometry is necessary in critically ill patients as poor agreement between measurements of pupil size collected using an automated device versus conventional clinical assessment has been reported, in particular for small pupil sizes [44]. Automated pupillometry can also provide some important prognostic information; in particular, the NPI was a good predictor of the development of intracranial hypertension or midline shift in brain-injured patients and provided an accurate identification of patients with extended post-anoxic brain injury [45–47]. However, no study has examined the role of automated pupillometry as a monitoring or predictive tool for CAR.

Why our hypothesis could not be confirmed by the results of this study? We included a heterogeneous population of critically ill patients; it is therefore possible that diseases inducing both an alteration in cerebral autoregulation and pupillary response would allow a better comparison on the monitoring tools while this would not be possible in others. As such, sepsis is frequently associated with brain dysfunction and impairment of cerebral autoregulation [12]. Few data are available on alterations in pupillary response using the automated pupillometry in these patients [48]; however, common pathways in brain and pupillary dysfunction, in particular of the cholinergic pathways, have been described in sepsis and might explain why we could observe a significant correlation only in this subgroup of patients. Nevertheless, the correlation was weak, as additional factors, such as sedative or analgesics, may significantly influence pupillary function in this setting as additional confounders [18, 19]. Moreover, most of NPI values ranged between 3.5 and 5.0, within the relatively normal ranges [27]. As such, it would be difficult to explore a possible correlation with another variable if only minimal changes in NPI were recorded. The lack of correlation between other pupillometry-derived variables, with a larger range of values, would suggest that automated pupillometry is not a reliable tool to predict impaired CAR in critically ill patients.

This study has some limitations to acknowledge. First, the number of patients included was relatively small in the two subgroups so that the statistical analysis was limited, and no further associative evaluation could be performed. However, it would be unlikely that adding more patients would have changed our conclusions. Second, we did not adjust our findings according to the use and/or total dose of sedatives or analgesics. Although this might have been of interest to eliminate potential bias, the use of automated pupillometry is of particular interest in those patients with an unreliable clinical examination (e.g., those under sedation) so that further statistical correction was not considered necessary. Also, the impact of other interesting variables, such as oxygenation, PaCO2, hemoglobin, and body temperature, on the relationship between pupillometry and autoregulation could not be further assessed because of the limited cohort. Third, automated pupillometry does not provide a continuous measurement of pupillary activity, while CAR assessment relies on a continuous recording of BP and FV for several minutes. Fourth, we did not assess the relationship between CAR or pupillometry and outcome; albeit of interest, one single measurement would not be informative in this setting. Further research using repeated measurements would be of interest in the future. Fifth, we dichotomized CAR as intact or impaired using a threshold suggested in previous studies; however, CAR is a continuous phenomenon, and a fixed Mxa value to define its function could have some limitations. Finally, NPI is based on an algorithm that incorporates several quantitative features of the pupillary light reflex; as such, the relationship of each of these components with the autonomic nervous system remains unknown.

## Conclusion

This study reported a weak association between the cerebral autoregulation and pupillometry-derived variables. These data also suggest that septic patients could be a population where pupillometry might give some information on the autoregulatory capacity of the brain.

## Data Availability

The datasets used and/or analyzed during the current study are available from the corresponding author on reasonable request.

## Abbreviations

ANS: Autonomic nervous System
APACHE: Acute Physiology and Chronic Health Evaluation
BP: Blood pressure
CAR: Cerebral autoregulation
CBF: Cerebral blood flow
CPP: Cerebral perfusion pressure
ECMO: Extracorporeal Membrane Oxygenation
FV: Blood flow velocity
ICU: Intensive care unit
MCA: Middle cerebral artery
Mxa: Mean flow index
MAP: Mean arterial pressure
NPI: Neurological Pupil Index©
PaCO_2_: Arterial pressure of carbon dioxide
PLR: Pupillary light reflex
PDMS: Patient data management system
ROC: Receiver operating characteristic
SABD: Sepsis-associated brain dysfunction
SAH: Subarachnoid hemorrhage
TBI: Traumatic brain injury
TCD: Transcranial Doppler

## References

[1] Singer M, Deutschman CS, Seymour CW, Shankar-Hari M, Annane D, Bauer M (2016). The third international consensus definitions for sepsis and septic shock (Sepsis-3). JAMA.

[2] Vincent JL, Rello J, Marshall J, Silva E, Anzueto A, Martin CD, Moreno R, Lipman J, Gomersall C, Sakr Y, Reinhart K. EPIC II Group of Investigators. International study of the prevalence and outcomes of infection in intensive care units. JAMA 2009; 302(21):2323-2329. 10.1001/jama.2009.1754 19952319

[3] Vincent JL, Sakr Y (2006). Sepsis in European intensive care units: results of the SOAP study. Crit Care Med.

[4] Kadri SS, Rhee C, Strich JR, Morales MK, Hohmann S, Menchaca J (2017). Estimating ten-year trends in septic shock incidence and mortality in United States academic medical centers using clinical data. Chest.

[5] Robba C, Crippa IA, Taccone FS (2018). Septic encephalopathy. Curr Neurol Neurosci Rep.

[6] Heming N, Mazeraud A, Verdonk F, Bozza FA, Chrétien F, Sharshar T (2017). Neuroanatomy of sepsis-associated encephalopathy. Crit Care.

[7] Taccone FS, Su F (2010). Cerebral microcirculation is impaired during sepsis: an experimental study. Crit Care.

[8] De Backer D, Cortes DO (2014). Pathophysiology of microcirculatory dysfunction and the pathogenesis of septic shock. Virulence..

[9] Ehler J, Barret LK, Taylor V, Groves M, Scaravilli F, Wittsock M (2017). Translational evidence for two distinct patterns of neuroaxonal injury in sepsis: a longitudinal, prospective translational study. Crit Care.

[10] Madhok DY, Vitt JR, Nguyen AT (2018). Overview of neurovascular physiology. Curr Neurol Neurosci Rep.

[11] Oddo M, Taccone FS (2015). How to monitor the brain in septic patients?. Minerva Anestesiol.

[12] Crippa IA, Subira C, Vincent JL, Fernandez RF, Hernandez SC, Zama Cavicchi F (2018). Impaired cerebral autoregulation is associated with brain dysfunction in patients with sepsis. Crit Care.

[13] Adam S, Kandelman S (2013). Sepsis-induced brain dysfunction. Exp Rev Anti Infect Therap.

[14] Schramm P, Klein KU, Falkenberg L, Berres M, Closhen D, Werhahn KJ (2012). Impaired cerebrovascular autoregulation in patients with severe sepsis and sepsis-associated delirium. Crit Care.

[15] Donnelly J, Budohoski KP, Smielewski P, Czosnyka M (2016). Regulation of the cerebral circulation: bedside assessment and clinical implications. Crit Care.

[16] Fantini S, Sassaroli A (2016). Cerebral blood flow and autoregulation: current measurement techniques and prospects for noninvasive optical methods. Neurophotonics..

[17] McBryde FD, Malpas SC, Paton JF (2017). Intracranial mechanisms for preserving brain blood flow in health and disease. Acta Physiol (Oxf).

[18] Taccone FS, Scolletta S (2012). Brain perfusion in sepsis. Curr Vasc Pharmacol.

[19] Smith J, Flower O, Tracey A, Johnson P (2020). A comparison of manual pupil examination versus an automated pupillometer in a specialised neurosciences intensive care unit. Aust Crit Care.

[20] Olson DM, Stutzman S, Saju C, Wilson M, Zhao W, Aiyagari V (2014). Interrater reliability of pupillary assessments. Neurocrit Care.

[21] Hall CA, Chilcott RP (2018). Eyeing up the future of the pupillary light reflex in neurodiagnostics. Diagnostics..

[22] Phillips SS (2018). Mueller CM.

[23] Joshi S, Li Y, Kalwani RM, Gold JI (2016). Relationships between pupil diameter and neuronal activity in the locus coeruleus, colliculi, and cingulate cortex. Neuron.

[24] Pinto L, Goard MJ, Estandian D, Xu M, Kwan AC, Lee SH (2013). Fast modulation of visual perception by basal forebrain cholinergic neurons. Nature Neurosc.

[25] Solari D, Miroz JP. Opening a window to the injury brain: non-invasive neuromonitoring with quantitative pupillometry. Ann Update Intensive Care Emerg Med. 2018:503–18.

[26] Olson DM, Fishel M (2016). The use of automated pupillometry in critical care. Crit Care Nurs Clin N Am.

[27] Olson DM, Stutzman SE. Establishing normative data for pupillometer assessment in neuroscience intensive care: the “END-PANIC” registry. 2017;49(4):251-254. 10.1097/JNN.0000000000000296 28661950

[28] Sorrentino E, Karol P (2011). Critical thresholds for transcranial Doppler indices of cerebral autoregulation in traumatic brain injury. Neurocrit Care.

[29] Lang EW, Mehdorn HM (2002). Continuous monitoring of cerebrovascular autoregulation: a validation study. J Neurol Neurosurg Psychiatry.

[30] Zeiler FA, Donnelly J (2017). Continuous autoregulatory indices derives from multi-modal monitoring: each one is not like the other. J Neurotrauma.

[31] Budohoski KP, Czosnyka M, Smielewski P, Kasprowicz M, Helmy A, Bulters D (2012). Impairment of cerebral autoregulation predicts delayed cerebral ischemia after subarachnoid hemorrhage: a prospective observational study. Stroke.

[32] Castro P, Serrador JM, Rocha I, Sorond F, Azevedo E (2017). Efficacy of cerebral autoregulation in early ischemic stroke predicts smaller infarcts and better outcome. Front Neurol.

[33] Sorrentino E, Diedler J, Kasprowicz M, Budohoski KP, Haubrich C, Smielewski P (2011). Critical thresholds for cerebrovascular reactivity after traumatic brain injury. Neurocrit Care.

[34] Pham P, Bindra J, Chuan A, Jaeger M, Aneman A (2015). Are changes in cerebrovascular autoregulation following cardiac arrest associated with neurological outcome? Results of a pilot study. Resuscitation.

[35] Kirkness CJ, Mitchell PH (2001). Cerebral autoregulation and outcome in acute brain injury. Biol Res Nurs.

[36] Sundgreen C, Larsen FS (2001). Autoregulation of cerebral blood flow in patients resuscitated from cardiac arrest. Stroke.

[37] Hu K, Peng CK, Czosnyka M, Zhao P, Novak V (2008). Nonlinear assessment of cerebral autoregulation from spontaneous blood pressure and cerebral blood flow fluctuations. Cardiovasc Eng.

[38] Kirkham SK, Craine RE (2001). A new mathematical model of dynamic cerebral autoregulation based on a flow dependent feedback mechanism. Physiol Meas.

[39] Schreiber SJ, Gottschalk S (2000). Assessment of blood flow velocity and diameter of the middle cerebral artery during the acetazolamide provocation test by use of transcranial Doppler sonography and MR imaging. Am J Neuroradiol.

[40] Czosnyka M, Smielewski P (2003). Continuous assessment of cerebral autoregulation: clinical and laboratory experience. Acta Neurochir Suppl.

[41] Taccone FS, Castanares-Zapatero D, Peres-Bota D, Vincent JL (2009). Berre’ J, Melot C. cerebral autoregulation is influenced by carbon dioxide levels in patients with septic shock. Neurocrit Care.

[42] Sekhon MS, Griesdale DE, Czosnyka M, Donnelly J, Liu X, Aries MJ (2015). The effect of red blood cell transfusion on cerebral autoregulation in patients with severe traumatic brain injury. Neurocrit Care.

[43] Lagi A, La Villa G (1997). Cerebral autoregulation in patients with cirrhosis and ascites. J Hepatol.

[44] Couret D, Boumaza D, Grisotto C, Triglia T, Pellegrini L, Ocquidant P (2016). Reliability of standard pupillometry practice in neurocritical care: an observational, double-blinded study. Crit Care.

[45] Chen J, Gombart Z (2011). Pupillary reactivity as an early indicator of increased intracranial pressure: the introduction of the neurological pupil index. Surg Neurol Int.

[46] Oddo M, Sandroni C, Citerio G, Miroz JP, Horn J, Rundgren M (2018). Quantitative versus standard pupillary light reflex for early prognostication in comatose cardiac arrest patients: an international prospective multicenter double-blinded study. Intensive Care Med.

[47] Jahns FP, Miroz JP, Messerer M, Daniel RT, Taccone FS, Eckert P (2019). Quantitative pupillometry for the monitoring of intracranial hypertension in patients with severe traumatic brain injury. Crit Care.

[48] Favre E, Bernini A, Morelli P, Pasquier J, Miroz JP, Abed-Maillard S (2020). Neuromonitoring of delirium with quantitative pupillometry in sedated mechanically ventilated critically ill patients. Crit Care.

